# An interpretable machine-learning model identifies obesity-related metabolic complications in children attending a tertiary obesity clinic

**DOI:** 10.3389/fped.2026.1969179

**Published:** 2026-09-23

**Authors:** Tingting Li, Nan Nan, Wenjie Wei, Ying Zhu, Jiayue Li, Lingzhi Zhang, Bingwu Wang, Zhenqiang Da, Jiaxin Chai, Cheng Wang, Xingwen Xie

**Affiliations:** 1 Department of Child Health Care, Gansu Province Maternity and Child Health Hospital, Lanzhou, Gansu, China; 2 School of Cyberspace Security, Gansu University of Political Science and Law, Lanzhou, Gansu, China; 3 Gansu Provincial Hospital of Traditional Chinese Medicine, Lanzhou, Gansu, China

**Keywords:** body composition, childhood obesity, LASSO, machine learning, metabolic complications, risk prediction

## Abstract

Childhood obesity-related metabolic complications are common in Northwest China, yet region-specific evidence and risk-stratification tools are lacking. In a single-visit cross-sectional study of 131 children (3–18 years) with overweight or obesity attending a Gansu maternal and child-care hospital, obesity-related metabolic complications (ORMC) were present in 31.3% (dyslipidaemia 16.0%, hyperuricaemia 9.2%, elevated blood pressure 7.6%). An interpretable model combining LASSO-selected adiposity measures (body mass index, neck circumference, body fat percentage) with penalised logistic regression achieved moderate, internally cross-validated discrimination (AUC 0.72, 95% CI 0.62-0.82) that awaits external validation, comparable to a random forest (0.73). Screen time was the strongest modifiable behavioural correlate. Routine metabolic screening and simple body-composition measures should be embedded in paediatric obesity clinics, with screen-time reduction as a counselling priority.

## 1 Introduction

Childhood obesity has become one of the most serious public-health challenges of the twenty-first century. Between 1990 and 2022 the global prevalence of obesity among school-aged children and adolescents increased from 1.7% to 6.9% in girls and from 2.1% to 9.3% in boys, affecting approximately 160 million young people worldwide (1, 2). In the United States, 16.9% of children and adolescents were already obese in 2011–2012 (3). China now carries the largest population of children with overweight or obesity of any country, driven by rapid nutrition transition, urbanisation, declining physical activity and expanding screen-based sedentary behaviour (4–6). The World Health Organization's Commission on Ending Childhood Obesity has called for urgent action, because obesity in childhood tracks into adulthood and predicts premature cardiometabolic disease (7–9).

Obesity-related metabolic disturbances are not confined to adults. Among children with obesity, insulin resistance, dyslipidaemia, elevated blood pressure, non-alcoholic fatty liver disease and impaired glucose tolerance are already common (10–13). In a landmark United States cohort, Weiss and colleagues demonstrated that the prevalence of the metabolic syndrome reached 50% in severely obese adolescents and rose with worsening obesity (11); systematic reviews report prevalences ranging from about 12% in overweight to over 60% in some obese cohorts, depending on the definition used (14, 15). Chinese data are consistent with this picture: in a representative Beijing sample, 27.6% of adolescents with obesity met International Diabetes Federation criteria for the metabolic syndrome vs. 0.2% of normal-weight peers (16), and national surveys confirm strong gradients of dyslipidaemia and hypertension across body-mass-index (BMI) categories (17). Hyperuricaemia, an increasingly recognised cardiometabolic risk marker, is likewise clustering in children with obesity in China (18). Early identification of children with obesity who already harbour metabolic complications is therefore a clinical priority, because lifestyle intervention is most effective before irreversible vascular and hepatic damage accrues (8, 10).

Risk stratification in paediatric obesity clinics remains suboptimal. BMI, although recommended by Chinese and international guidelines as the primary screening index (19–21), does not capture fat distribution or body composition; central and visceral adiposity, approximated by waist-to-height ratio (WHtR), neck circumference or bioimpedance-derived body fat percentage, may carry additional metabolic information (10, 19). At the same time, machine-learning methods—penalised regression such as the least absolute shrinkage and selection operator (LASSO) (22) and ensemble learners such as random forests (23)—offer a principled way to combine correlated anthropometric, behavioural and familial variables into parsimonious prediction models, and have begun to show promise in paediatric metabolic research (10). However, most published models derive from large metropolitan cohorts in eastern China or from high-income countries; evidence from the less developed northwestern provinces, where dietary patterns, vitamin D status and health resources differ markedly, is essentially absent.

We therefore analysed a consecutive single-visit cross-sectional sample of children and adolescents with overweight or obesity attending Gansu Province Maternity and Child Health Hospital in Lanzhou, Northwest China. Our objectives were (i) to quantify the prevalence of obesity-related metabolic complications (ORMC) and their components, and (ii) to develop and internally validate an interpretable machine-learning model—combining LASSO feature selection, multivariable logistic regression and a random-forest benchmark—that identifies children at high risk of ORMC from routinely available clinical, behavioural and familial information. Because each participant contributed data from a single study visit with no follow-up, all analyses are cross-sectional and causal inference is not implied.

## 2 Methods

### 2.1 Study design and participants

Design. This was a single-centre, single-visit cross-sectional study, defined operationally as a study in which each participant contributed exposure, mediator and outcome data at one and the same study visit, with no follow-up and no longitudinal ascertainment of events. The design therefore supports estimation of prevalence and cross-sectional associations, not incidence or causal temporal sequences; the term “long-term” does not apply to this study.

Setting and recruitment. Participants were recruited consecutively from the child health/weight-management outpatient clinic of Gansu Province Maternity and Child Health Hospital, Lanzhou, Northwest China, over a fixed 14-month enrolment window from 1 May 2025 to 30 June 2026. The clinic is the tertiary referral paediatric obesity service of the only provincial maternal and child-health institution in Gansu, accepts self- and primary-care referrals from across the province, and serves an ethnically and socio-economically diverse catchment. On each clinic day during the enrolment window, the attending physician invited every consecutive patient who met the eligibility criteria to participate; no implicit or explicit selection beyond the criteria below was applied.

Eligibility criteria. Inclusion criteria were (i) age 3–18 years at the time of the visit; (ii) a clinical diagnosis of overweight or obesity assigned by the attending physician, classified by the Chinese national screening standard for school-aged children and adolescents (WS/T 586—2018) (20, 24) and complemented by the age- and sex-specific BMI cut-offs recommended by the Chinese expert consensus on the diagnosis, assessment and management of childhood obesity (19); (iii) the child and the accompanying parent/guardian were willing to undergo the study measurements and to complete the structured questionnaire. Exclusion criteria were (i) secondary (endocrine, genetic or drug-induced) obesity; (ii) ongoing treatment for an acute illness or an established diagnosis of type 1 diabetes mellitus, monogenic obesity or a syndromic obesity phenotype; and (iii) inability to complete the study procedures for any reason. The number of children excluded for secondary obesity (*n* = 0) was documented from the screening log.

Sample. The sample comprised all eligible children registered during the 14-month enrolment window; no *a priori* sample-size calculation was performed and no external control group was assembled. Each child contributed data from a single study visit; there was no follow-up visit, no repeated measurement, and no longitudinal ascertainment of outcomes. All data were abstracted from the structured clinic registry, de-identified before analysis, and stored on an encrypted institutional server accessible only to the principal investigator.

### 2.2 Anthropometry and body composition

Height and weight were measured with a calibrated stadiometer and electronic scale with participants in light clothing; BMI was computed as weight (kg)/height (m)^2^. Waist, hip, neck and chest circumferences were measured with a non-elastic tape; WHtR was calculated as waist circumference (cm)/height (cm). Body fat percentage, visceral fat level and skeletal muscle mass were estimated by segmental bioelectrical impedance analysis. Birth weight, parental heights and family history of hypertension, diabetes, hyperuricaemia and fatty liver were reported by parents or guardians. Implausible entries (e.g., values outside physiological ranges or obvious unit errors) were set to missing following pre-specified cleaning rules detailed in the analysis code.

### 2.3 Laboratory measurements and outcome definition

Fasting venous blood was assayed for triglycerides (TG), total cholesterol (TC), high- and low-density lipoprotein cholesterol (HDL-C, LDL-C), fasting plasma glucose (FPG), uric acid and 25-hydroxyvitamin D (25(OH)D) in the hospital laboratory. Resting blood pressure was measured with an appropriately sized cuff. The primary endpoint, ORMC, was defined as the presence of at least one of the following, using cut-offs consistent with the Chinese expert consensus (19), the harmonised international metabolic-syndrome criteria (25), the Chinese paediatric metabolic-syndrome evidence (16) and the 2011 National Heart, Lung, and Blood Institute integrated guidelines (26): (i) dyslipidaemia (TG ≥1.47 mmol/L, HDL-C < 1.03 mmol/L, LDL-C ≥ 3.36 mmol/L or TC ≥5.18 mmol/L); (ii) hyperuricaemia (serum uric acid ≥360 µmol/L) (18); (iii) impaired fasting glucose (FPG ≥5.6 mmol/L); (iv) elevated blood pressure [systolic ≥120 or diastolic ≥80 mmHg, the rapid screening threshold recommended by the Chinese consensus and concordant with the 2017 American Academy of Pediatrics guideline for adolescents (19, 27)]; or (v) physician-documented insulin resistance or fatty liver. Vitamin D deficiency was defined as 25(OH)D < 20 ng/mL (28) and analysed descriptively. These conditions were combined into a single binary endpoint because they share a common adiposity- and insulin-resistance-driven pathophysiology, co-cluster within individuals as components of the paediatric metabolic-syndrome spectrum (11, 25), and each independently prompts further testing or management; a composite therefore captures the cumulative metabolic burden that triggers clinical action and increases the number of informative events for modelling. The fixed 120/80 mmHg threshold was adopted deliberately as a pragmatic screening cut-off rather than a diagnostic criterion: it permits rapid, uniform classification during a single clinic visit without age-, sex- and height-percentile look-up tables; for adolescents (≥13 years) it coincides with the 2017 American Academy of Pediatrics definition of elevated blood pressure (27); and in younger children it approximates the 90th percentile for a child of median height, functioning as a sensitive screening threshold (27). Children screening positive would require confirmatory percentile-based classification and repeated measurements on separate occasions before any diagnosis of hypertension (27); the classification implications of this simplification are addressed in the Limitations.

### 2.4 Lifestyle and family-environment assessment

A structured parent-report questionnaire captured meal regularity, frequencies of sweet foods, fried foods and bedtime snacks, daily physical activity, organised sports participation, recreational screen time, sleep duration, staying up late, parental weight status, family high-fat/high-sugar dietary pattern, and parental awareness of obesity-related harms. Ordinal items were coded monotonically (higher scores=less favourable behaviour, except physical activity); binary items were coded 0/1. Recreational screen time—leisure-time use of electronic devices (phones, tablets, computers, television), excluding educational use—was reported by parents in four ordinal categories (≤1, 1–2, 2–3 and >3 h/day) and coded 0–3. Weekday and weekend use were not recorded separately, and the questionnaire was a study-specific instrument that had not been formally validated, which we acknowledge as a measurement limitation.

### 2.5 Statistical analysis and machine learning

Continuous variables are summarised as mean ± standard deviation (SD) or median (interquartile range) and compared between children with and without ORMC using the Mann–Whitney U test; categorical variables were compared using the *χ*^2^ test or Fisher's exact test. Component prevalences are reported with Wilson 95% confidence intervals (CI). Candidate predictors (*n* = 22; Table 1) were standardised (z-scores). Predictor missingness was low (≤14.5% for any single predictor; per-variable values in Supplementary Table 2) and was imputed with the median (continuous) or mode (categorical); these simple, transparent imputations were chosen because few predictors were affected, the mechanism was plausibly missing at random, and most affected variables were skewed or ordinal, for which median/mode imputation is robust and avoids additional model-based assumptions. A complete-case sensitivity analysis (*n* = 95) evaluated the impact of this choice. In contrast, the laboratory assays defining the ORMC components showed higher missingness (up to 44.3% for fasting lipids; Supplementary Table 2); these were not imputed but were handled through component-availability rules, whereby a component was scored only when the relevant assay was available and a child was classifiable for ORMC when at least one component could be assessed. As a sensitivity analysis, multiple imputation by chained equations gave essentially the same cross-validated discrimination (AUC 0.73) as median/mode imputation (AUC 0.72), supporting the robustness of the primary imputation strategy.

**Table 1:** Characteristics of the study population by ORMC status.

Variable	ORMC (*n* = 41)	No ORMC (*n* = 90)	P
Age (years)	10.0 (8.0–12.0)	8.0 (6.0–10.0)	<0.001
Male sex	19 (46.3%)	28 (31.1%)	0.092
BMI (kg/m^2^)	24.2 (21.0–27.5)	20.4 (18.4–21.9)	<0.001
Waist-to-height ratio	0.55 (0.51–0.60)	0.51 (0.48–0.55)	0.003
Neck circumference (cm)	31.2 (29.0–34.0)	28.5 (27.2–30.0)	<0.001
Body fat percentage (%)	37.0 (30.6–41.6)	29.5 (24.5–33.4)	<0.001
Visceral fat level	9.0 (5.0–12.0)	4.0 (2.0–6.0)	<0.001
Birth weight ≥4 kg	2 (4.9%)	7 (7.8%)	0.719
Only child	12 (29.3%)	31 (34.4%)	0.559
Family history of hypertension	16 (39.0%)	25 (27.8%)	0.198
Family history of diabetes	8 (19.5%)	22 (24.4%)	0.533
Sweet food frequency (0–3)	1.0 (1.0–2.0)	1.0 (1.0–2.0)	0.290
Fried food frequency (0–3)	1.0 (1.0–1.0)	1.0 (1.0–1.0)	0.548
Bedtime snack frequency (0–3)	1.0 (0.0–1.0)	0.0 (0.0–1.0)	0.833
Daily physical activity (0–4)	2.0 (1.0–3.0)	2.0 (2.0–3.0)	0.686
Screen time (0–3)	1.0 (0.0–1.0)	0.0 (0.0–1.0)	0.031
Short sleep duration (0–2)	1.0 (1.0–2.0)	1.0 (1.0–1.0)	0.112
Staying up late (0–3)	2.0 (1.0–3.0)	1.0 (1.0–2.0)	0.058
Irregular meals (0–2)	0.0 (0.0–1.0)	0.0 (0.0–1.0)	0.608
Family high-fat/high-sugar diet (0–3)	1.0 (1.0–1.0)	1.0 (1.0–1.0)	0.057
Parental awareness of obesity harms (0–3)	1.0 (1.0–2.0)	1.0 (1.0–2.0)	0.074
Both parents overweight/obese	17 (41.5%)	26 (28.9%)	0.155

Data are median (interquartile range) or *n* (%). *P* values from Mann–Whitney U test (continuous) or *χ*^2^/Fisher's exact test (categorical). ORMC, obesity-related metabolic complication.

Univariable logistic regression estimated the odds ratio (OR) of ORMC per 1-SD increment of each predictor. Feature selection then used LASSO-penalised logistic regression, which minimises the penalised negative log-likelihood.

$$\beta^{\wedge }=argmin\left\{-\left(1/n\right)\Sigma_{i}\left[y_{i}\left(\beta_{0}+x_{i}^{T}\beta \right)-log\left(1+exp\left(\beta_{0}+x_{i}^{T}\beta \right)\right)\right]+\lambda \|\beta {\|}_{1}\right\}$$

With *λ* chosen by 10-fold cross-validation under the one-standard-error (1-SE) rule to favour parsimony (22). Variables retained by LASSO entered a multivariable logistic model, logit{P(Y = 1 | x)} = *β*₀ + *β*ᵀx, reported as adjusted ORs with 95% CIs; calibration was assessed with the Hosmer–Lemeshow test (29).

As a non-linear benchmark, a random-forest classifier (23) with 1,000 trees, class-balanced weighting and a minimum leaf size of five was fitted; the forest estimates P(Y = 1 | x) by the majority vote of B bootstrapped decision trees, $\hat{p}$(x) = (1/B) *Σ*_b T_b(x), and variable importance was quantified by permutation importance (mean decrease in AUC over 50 permutations). Discrimination of both models was estimated by out-of-fold predictions from 10-times-repeated stratified 5-fold cross-validation, with 95% CIs from 2,000 bootstrap resamples. Univariable screening was treated as hypothesis-generating and was not adjusted for multiplicity; inference was based on the penalised, internally validated models. All analyses used Python 3.13 with scikit-learn 1.x (30) and statsmodels; two-sided *P* < 0.05 was considered significant. The complete, annotated analysis script accompanies this article to ensure full reproducibility. Throughout, “machine learning” is used in its broad sense of supervised statistical learning: the primary model is LASSO-penalised logistic regression—a regularised generalised linear model chosen for interpretability—with a random forest included only as a non-linear benchmark, rather than a complex “black-box” artificial-intelligence system.

### 2.6 Ethics

The study protocol was approved by the Ethics Committee of Gansu Province Maternity and Child Health Hospital (approval No. 2024-GSFY-083) and was conducted in accordance with the Declaration of Helsinki and the CIOMS International Ethical Guidelines for Health-related Research Involving Humans. Written informed consent and, where required, written informed assent were obtained age-appropriately as follows: for children aged 3–6 years, written informed consent was obtained from a parent or legal guardian, with verbal age-appropriate information given to the child; for children aged 7–11 years, written informed consent was obtained from a parent or legal guardian and a separately written, age-appropriate informed assent form was signed by the child; and for adolescents aged 12–18 years, written informed consent was obtained from the adolescent themselves in addition to written informed consent from a parent or legal guardian (i.e., two independent written signatures). All consents and assents were obtained before any study procedure, the participants were explicitly informed of the voluntary nature of participation and the right to withdraw without giving a reason and without affecting routine clinical care, and no identifying data left the hospital.

## 3 Results

### 3.1 Cohort characteristics

Of 131 children enrolled (84 girls, 64.1%; 47 boys, 35.9%), mean age was 8.6 ± 3.1 years (range 3–18; Figure 1A). Mean BMI was 21.9 ± 4.1 kg/m^2^ (range 16.3–41.7), mean WHtR was 0.52, and mean bioimpedance body fat percentage was 30.8%. Family history was notable for hypertension in 31.3% and diabetes in 22.9% of children; 79.4% had at least one parent with overweight or obesity (father 67.9%, mother 44.3%).

**Figure 1:**
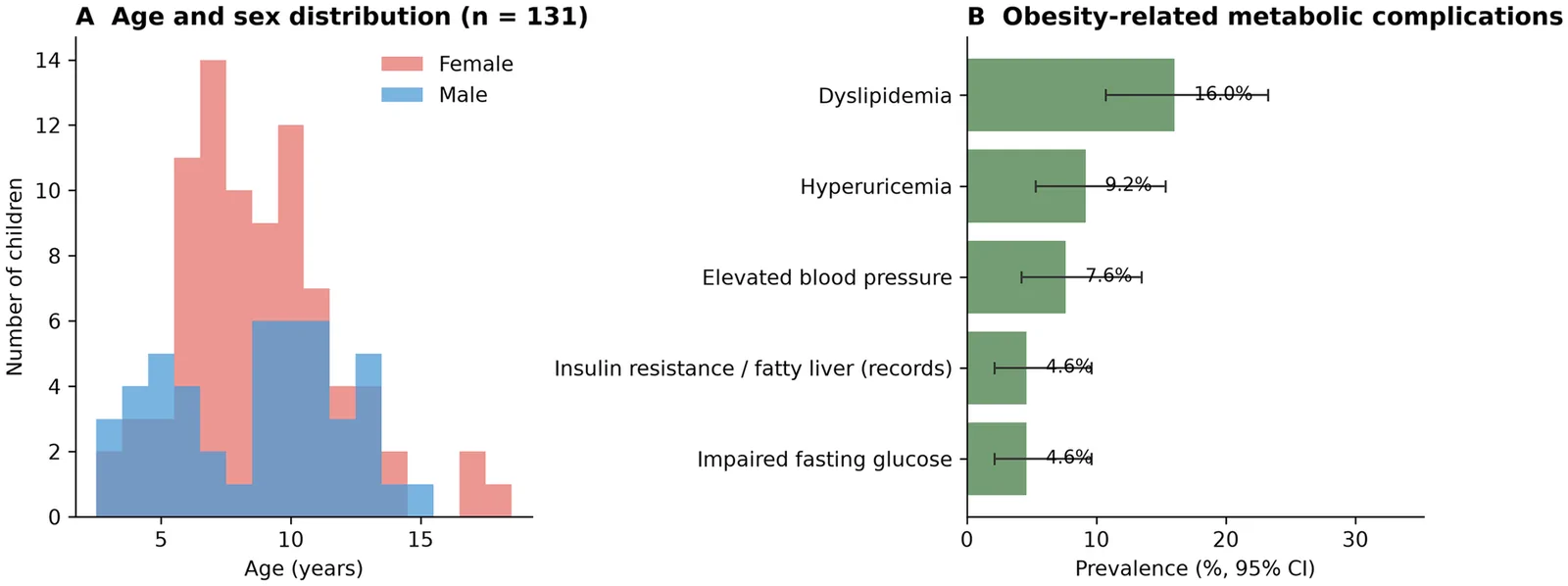
**(A)** Age and sex distribution of the cohort (*n* = 131). **(B)** Prevalence of obesity-related metabolic complication components with Wilson 95% confidence intervals.

### 3.2 Prevalence of obesity-related metabolic complications

ORMC was present in 41 of 131 children (31.3%, 95% CI 24.0–39.7%; Figure 1B and Table 2). Dyslipidaemia was the most frequent component (16.0%, 95% CI 10.7–23.3), followed by hyperuricaemia (9.2%, 5.3–15.3), elevated blood pressure (7.6%, 4.2–13.5), impaired fasting glucose (4.6%, 2.1–9.6) and physician-documented insulin resistance or fatty liver (4.6%, 2.1–9.6). Among the 89 children with available 25(OH)D measurements, vitamin D deficiency affected 29.2%. Ten children (7.6% of the cohort; about one in four of those with ORMC) had two or more simultaneous metabolic complications. ORMC prevalence rose steeply with age, from 11.4% at 3–6 years to 32.4% at 7–11 years and 60.0% at 12–18 years.

**Table 2:** Prevalence of ORMC and its components (wilson 95% CI).

Component	Events	N	Prevalence, % (95% CI)
Dyslipidemia	21	131	16.0 (10.7–23.3)
Hyperuricemia	12	131	9.2 (5.3–15.3)
Impaired fasting glucose	6	131	4.6 (2.1–9.6)
Elevated blood pressure	10	131	7.6 (4.2–13.5)
Insulin resistance/fatty liver (records)	6	131	4.6 (2.1–9.6)

### 3.3 Comparison by ORMC status

Children with ORMC were older (median 10 vs. 8 years, *P* < 0.001) and had markedly higher adiposity: BMI 24.2 vs. 20.4 kg/m^2^ (*P* < 0.001), neck circumference 31.2 vs. 28.5 cm (*P* < 0.001), body fat percentage 37.0% vs. 29.5% (*P* < 0.001), visceral fat level 9 vs. 4 (*P* < 0.001) and higher WHtR (*P* = 0.003) (Table 1). Recreational screen time was greater in the ORMC group (*P* = 0.031); dietary frequencies, sleep, family history and parental weight status did not differ significantly.

### 3.4 Univariable associations

In univariable logistic models (Table 3), the strongest per-SD associations with ORMC were observed for BMI (OR 3.44, 95% CI 2.01–5.89), neck circumference (OR 2.80, 1.76–4.45), body fat percentage (OR 2.68, 1.65–4.36), visceral fat level (OR 2.60, 1.63–4.13), age (OR 2.21, 1.44–3.40), WHtR (OR 1.77, 1.14–2.74) and screen time (OR 1.55, 1.07–2.23).

**Table 3:** Univariable logistic regression for ORMC (per 1-SD increment for continuous/ordinal predictors).

Variable	OR (95% CI)	P
Age (years)	2.21 (1.44–3.40)	<0.001
Male sex	1.37 (0.95–1.97)	0.094
BMI (kg/m^2^)	3.44 (2.01–5.89)	<0.001
Waist-to-height ratio	1.77 (1.14–2.74)	0.012
Neck circumference (cm)	2.80 (1.76–4.45)	<0.001
Body fat percentage (%)	2.68 (1.65–4.36)	<0.001
Visceral fat level	2.60 (1.63–4.13)	<0.001
Birth weight ≥4 kg	0.88 (0.58–1.33)	0.546
Only child	0.89 (0.61–1.30)	0.559
Family history of hypertension	1.27 (0.88–1.82)	0.200
Family history of diabetes	0.89 (0.60–1.30)	0.534
Sweet food frequency (0–3)	1.18 (0.82–1.71)	0.368
Fried food frequency (0–3)	1.09 (0.76–1.58)	0.633
Bedtime snack frequency (0–3)	0.91 (0.62–1.33)	0.627
Daily physical activity (0–4)	0.96 (0.66–1.39)	0.836
Screen time (0–3)	1.55 (1.07–2.23)	0.019
Short sleep duration (0–2)	1.34 (0.92–1.96)	0.130
Staying up late (0–3)	1.44 (0.98–2.10)	0.061
Irregular meals (0–2)	1.10 (0.76–1.58)	0.620
Family high-fat/high-sugar diet (0–3)	1.43 (0.98–2.09)	0.064
Parental awareness of obesity harms (0–3)	1.43 (0.98–2.06)	0.061
Both parents overweight/obese	1.30 (0.90–1.87)	0.157

### 3.5 LASSO selection and multivariable model

LASSO with the 1-SE rule retained three predictors—BMI, neck circumference and body fat percentage (Figure 2)—which together capture the adiposity domain while minimising collinearity-driven overfitting. In the multivariable model (Table 4), adjusted ORs per SD were 1.87 (95% CI 0.95–3.70, *P* = 0.072) for neck circumference, 1.71 (0.93–3.12, *P* = 0.082) for body fat percentage and 1.56 (0.64–3.78, *P* = 0.324) for BMI; the attenuation of BMI reflects shared variance with the two composition-specific measures. The Hosmer–Lemeshow test was non-significant (*χ*^2^ = 9.02, *P* = 0.341); however, given the out-of-fold calibration slope of 2.22 and intercept of −0.49 (Figure 3), this should not be read as evidence of adequate calibration—predicted probabilities were under-dispersed and calibration was imperfect, so the model requires recalibration before any clinical use.

**Figure 2:**
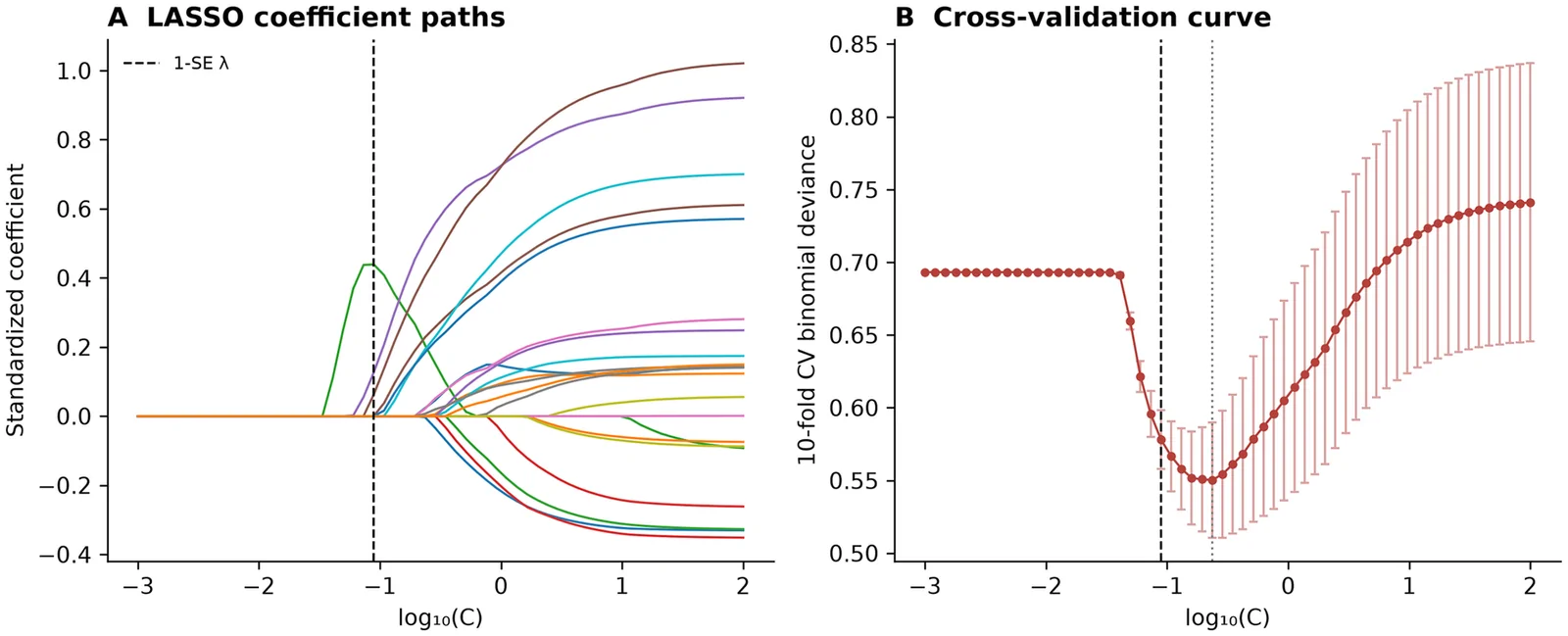
LASSO feature selection. **(A)** Standardised coefficient paths across the regularisation grid. **(B)** Ten-fold cross-validated binomial deviance (mean ± SE); dotted line, minimum; dashed line, one-standard-error solution retaining BMI, neck circumference and body fat percentage.

**Table 4:** Multivariable logistic model after LASSO selection (per 1-SD increment; hosmer–lemeshow *χ*^2^ = 9.02, *P* = 0.341).

Variable	Adjusted OR (95% CI)	P
BMI (kg/m^2^)	1.56 (0.64–3.78)	0.324
Neck circumference (cm)	1.87 (0.95–3.70)	0.072
Body fat percentage (%)	1.71 (0.93–3.12)	0.082

**Figure 3:**
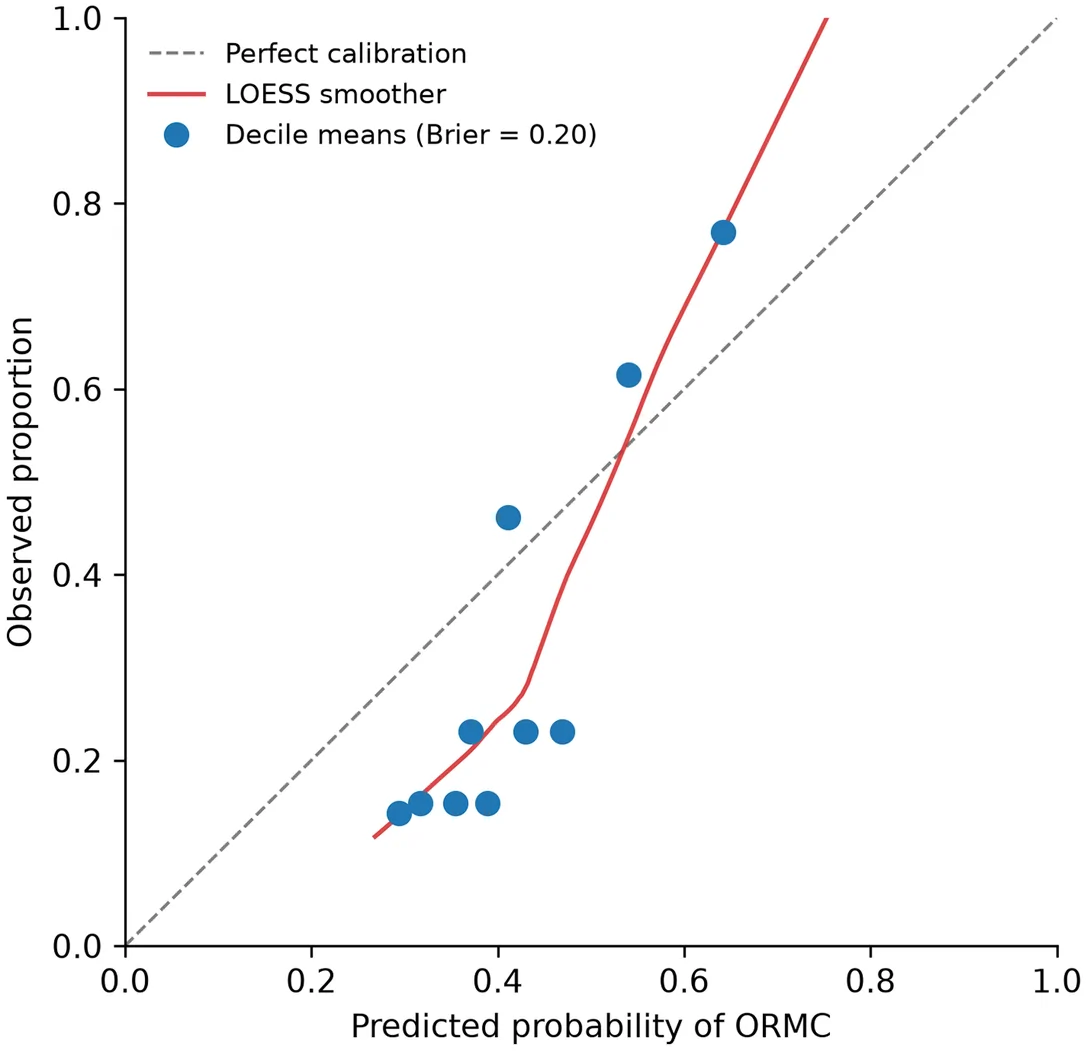
Calibration of the LASSO logistic model from out-of-fold predictions. The dashed line denotes perfect calibration; points are decile means of predicted vs. observed risk and the red curve is a LOESS smoother (Brier score 0.20; calibration intercept −0.49, slope 2.22).

### 3.6 Model discrimination and internal validation

Cross-validated discrimination was moderate and consistent across learners (Table 5, Figure 4): AUC 0.72 (95% CI 0.62–0.82) for the LASSO logistic model and 0.73 (0.61–0.83) for the random forest. The complete-case sensitivity analysis (*n* = 95) yielded AUC 0.70 (0.58–0.82), indicating robustness to the imputation strategy. Random-forest permutation importance ranked body fat percentage and neck circumference as the two most informative variables, ahead of WHtR, age, visceral fat and BMI, with screen time among the highest-ranking behavioural features (Figure 5). The Hosmer–Lemeshow test did not reject adequate fit (*P* = 0.341), but graphical calibration from out-of-fold predictions (Figure 3) showed a calibration slope of 2.22 and an intercept of −0.49; together with the small number of events, this indicates imperfect calibration with under-dispersed predicted probabilities, so the model should not be described as satisfactorily calibrated and requires recalibration and external validation before any clinical use. At the Youden-optimal predicted-probability threshold (0.51), the model showed sensitivity 0.46, specificity 0.91, positive predictive value 0.70 and negative predictive value 0.79. Decision-curve analysis (Figure 6) suggested a positive net benefit across a broad range of threshold probabilities; however, given the small sample, only 41 outcome events and the absence of external validation, this analysis is exploratory and hypothesis-generating and does not by itself establish clinical utility.

**Table 5:** Model discrimination under repeated stratified cross-validation with bootstrap confidence intervals.

Model	Cross-validated AUC (95% CI)
LASSO logistic regression (primary)	0.72 (0.62–0.82)
Random forest (benchmark)	0.73 (0.61–0.83)
LASSO logistic regression, complete-case sensitivity (*n* = 95)	0.70 (0.58–0.82)

**Figure 4:**
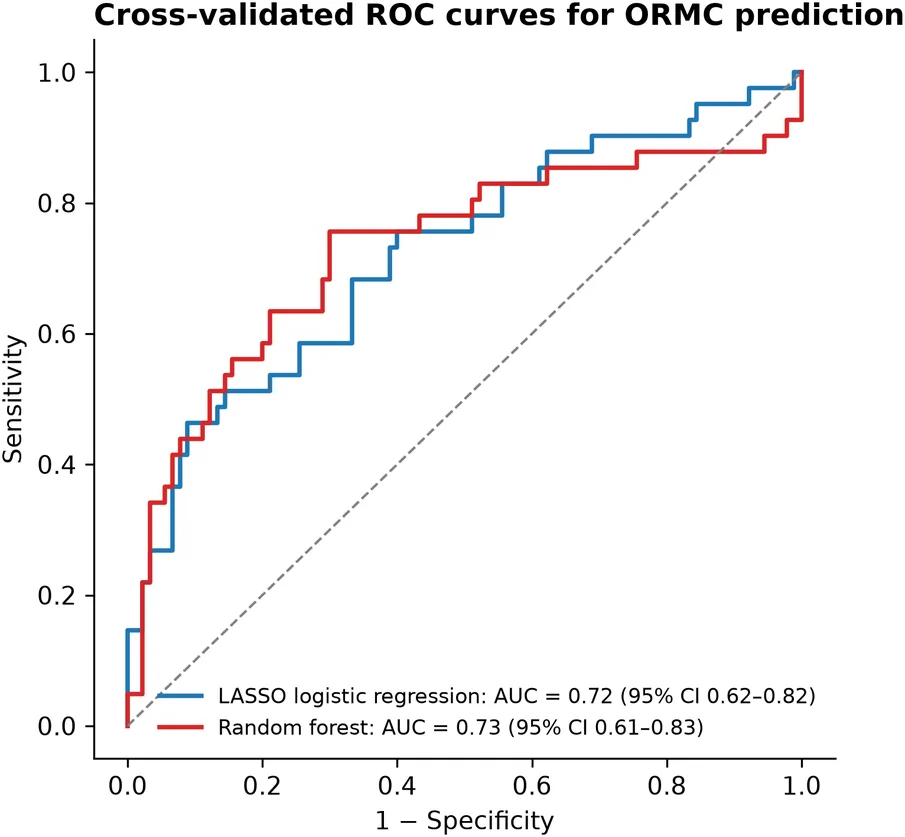
Cross-validated receiver operating characteristic curves for the LASSO logistic model and the random forest; areas under the curve with bootstrap 95% confidence intervals.

**Figure 5:**
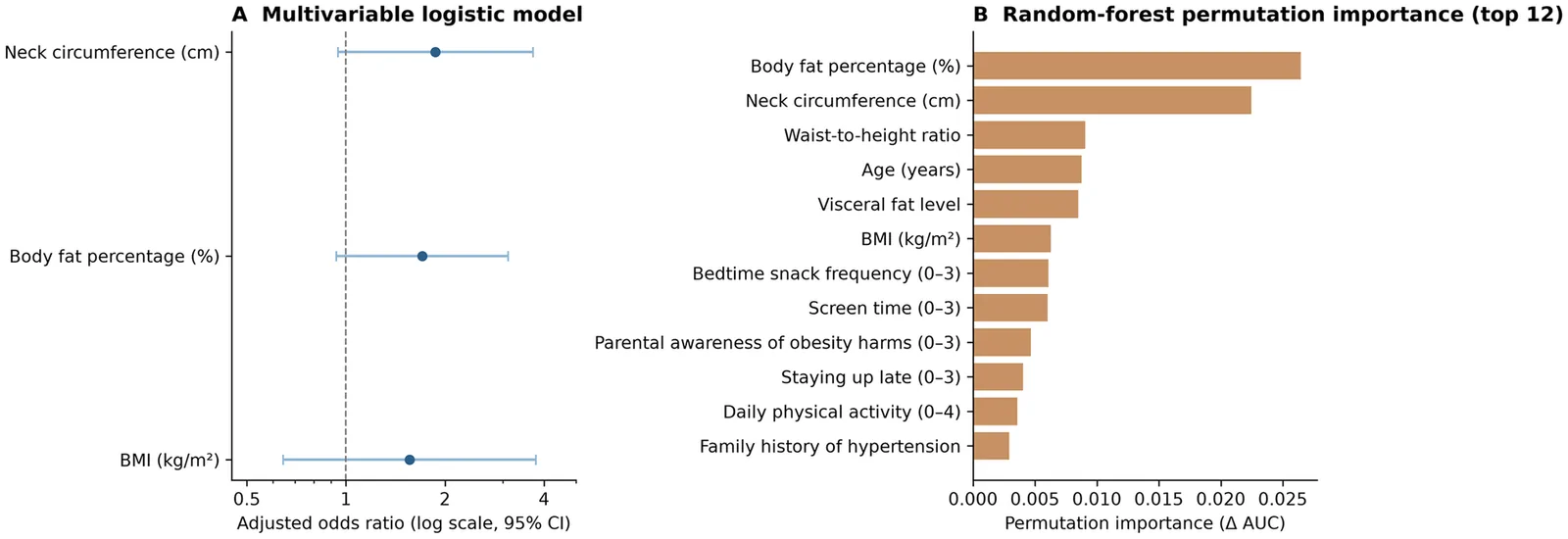
**(A)** adjusted odds ratios (95% CI, log scale) from the multivariable logistic model. **(B)** Random-forest permutation importance (mean decrease in AUC over 50 permutations), top 12 predictors.

**Figure 6:**
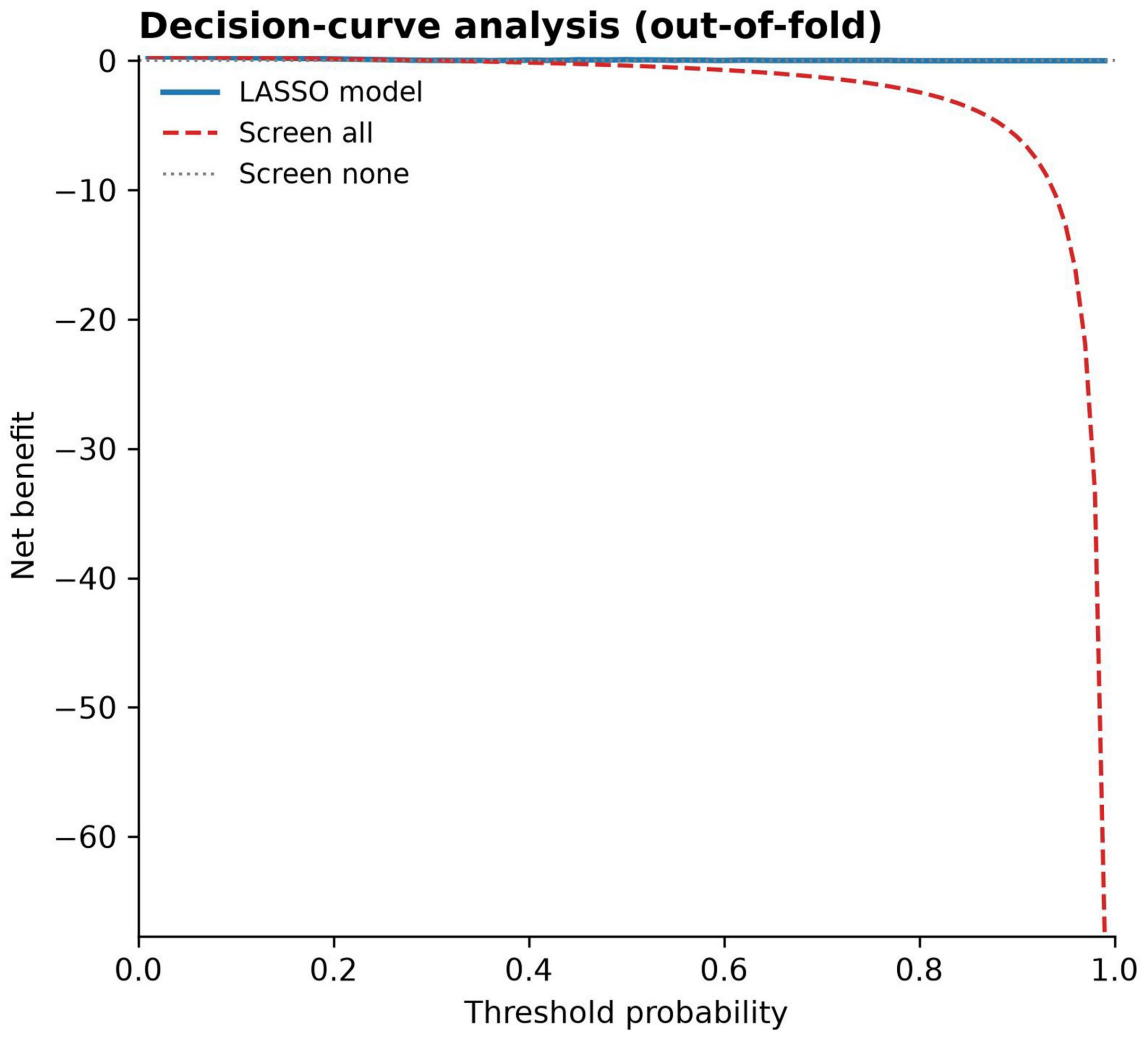
Exploratory decision-curve analysis of the LASSO logistic model from out-of-fold predictions (41 events; no external validation). Net benefit is shown across threshold probabilities for the model, a screen-all strategy and a screen-none strategy; the results are hypothesis-generating.

## 4 Discussion

### 4.1 Principal findings

In this single-visit cross-sectional sample of children with overweight or obesity from Northwest China, almost one in three children already presented at least one obesity-related metabolic complication, and one in six had dyslipidaemia. Adiposity burden—measured jointly by BMI, neck circumference and bioimpedance body fat—dominated prediction; an interpretable LASSO logistic model and a random forest achieved moderate, honestly cross-validated discrimination (AUC ≈ 0.72–0.73). Screen time emerged as the strongest modifiable behavioural correlate, and vitamin D deficiency was common.

### 4.2 Comparison with previous work

Our composite endpoint prevalence (31.3%) is broadly consistent with international estimates of metabolic-syndrome-component clustering in children with obesity (11, 14, 15) and with Chinese data: Chen et al. reported metabolic syndrome in 27.6% of Beijing adolescents with obesity (16), and Li et al. found pronounced clustering of dyslipidaemia and hypertension in overweight and obese Chinese children (17). The high burden we observed in a relatively young sample (mean age 8.6 years) echoes the warning of the NCD Risk Factor Collaboration that the obesity epidemic is expanding across all generations, including the youngest (1, 31). The prominence of hyperuricaemia (9.2%) in our cohort is consistent with Chinese clinical guidance on hyperuricaemia in the context of childhood obesity (18) and supports including uric acid in routine obesity-clinic panels. Vitamin D deficiency in 29.2% of tested children is plausible for a high-latitude northwestern population with limited winter sunlight and adds a region-specific nutritional dimension rarely reported in eastern coastal cohorts (28).

Rationale and implications of the composite endpoint. We combined dyslipidaemia, hyperuricaemia, elevated blood pressure, impaired fasting glucose and physician-documented insulin resistance or fatty liver into a single obesity-related metabolic complication (ORMC) endpoint for three reasons. First, these conditions share a common adiposity- and insulin-resistance-driven pathophysiology and co-cluster within individuals as components of the paediatric metabolic-syndrome spectrum (11, 16, 25), so a composite reflects the cumulative cardiometabolic burden rather than any single, somewhat arbitrary, cut-off. Second, each component independently prompts further testing or lifestyle or pharmacological management in obesity clinics, so identifying children with any complication is the clinically actionable question. Third, pooling components increased the number of informative events, improving the stability of an exploratory model in a modest sample. We acknowledge the trade-offs: the components are heterogeneous in severity and reversibility, their predictors may differ, and a composite can dilute component-specific signals and mask which condition drives a given prediction. ORMC should therefore be read as a pragmatic screening construct rather than a unified disease entity, and component-specific models would be preferable in larger samples.

### 4.3 Interpretation of the predictive findings

A key observation is methodological as much as clinical: once neck circumference and body fat percentage entered the LASSO model, BMI lost its independent association. Neck circumference indexes upper-body subcutaneous fat and has repeatedly outperformed BMI in paediatric cardiometabolic risk stratification; bioimpedance body fat percentage directly estimates adiposity rather than weight-for-height. Their priority in both the penalised linear model and the random-forest importance ranking supports the Chinese expert-consensus recommendation that body-composition and central-obesity indices should complement BMI in obesity assessment (10, 19). Screen time, the only behavioural variable surviving univariable screening, is a recognised determinant in Chinese children (4–6) and, being modifiable, is an actionable counselling target. The modest absolute AUCs are expected for a single-visit, routinely collected feature set and are reported with optimistic-bias control (repeated cross-validation plus bootstrap intervals), which we consider a strength relative to split-sample claims in smaller studies. Finally, the prominence of BMI, neck circumference and body fat percentage should be read as predictive importance rather than confirmed independent statistical association: none reached conventional significance in the multivariable model (adjusted *P* = 0.07–0.32), because the 41 events limited power and the three adiposity measures share substantial variance. LASSO retention and random-forest permutation importance quantify how much a variable improves out-of-sample prediction, which is conceptually distinct from hypothesis testing, and the wide confidence intervals caution against causal or strongly inferential interpretation of any single coefficient.

### 4.4 Strengths and limitations

Strengths include comprehensive phenotyping (anthropometry, bioimpedance, fasting biochemistry, blood pressure and structured behavioural assessment), a clinically meaningful composite endpoint, penalised and ensemble machine-learning methods with rigorous internal validation, sensitivity analysis under complete cases, and full code availability for reproducibility.

Several limitations temper interpretation. First, the analysis rests on only 131 participants and 41 outcome events; with three retained predictors the events-per-variable ratio is modest (∼14), and although LASSO with the 1-SE rule and repeated cross-validation were used to curb overfitting, coefficient estimates and apparent performance may still be unstable, so the model should be regarded as hypothesis-generating rather than ready for clinical application. Second, the model was developed and evaluated in a single tertiary obesity clinic in Northwest China; internal cross-validation cannot replace external validation, so its transportability and generalisability to other settings, populations and case-mixes remain uncertain, and any conclusions about clinical implementation are correspondingly cautious. Third, biochemical measurements were incomplete (up to 44.3% missing for fasting lipids; per-variable values in Supplementary Table 2), requiring median/mode imputation for the few affected predictors and component-availability rules for the outcome; although a complete-case sensitivity analysis gave similar discrimination (AUC 0.70), residual bias from informative missingness cannot be excluded, and glycated haemoglobin was largely unavailable and could not be modelled. Fourth, lifestyle data were parent-reported and subject to recall and social-desirability bias; sleep insufficiency was coded with a uniform duration threshold rather than age-specific recommendations, which may have misclassified sleep sufficiency particularly in adolescents. Fifth, the sample included more girls than boys (64.1% female), which may reflect referral patterns and could limit generalisability to boys, although sex was not a significant predictor of ORMC. Sixth, blood-pressure classification used a pragmatic single-occasion screening threshold rather than full percentile-based ambulatory confirmation (27), and pubertal (Tanner) stage was not systematically ascertained across the 3–18-year range; because puberty strongly influences insulin resistance, lipids, blood pressure and body composition, the absence of pubertal-staging data is an important limitation that precluded adjustment. Finally, the cross-sectional design precludes causal or temporal inference. Moreover, an assessment of missingness indicated that the incomplete laboratory data were not missing completely at random: children with available fasting lipids were older and had higher BMI and a higher prevalence of ORMC than those without (all *P* < 0.05), so component prevalences—dyslipidaemia in particular—may be underestimated, an additional source of bias. Relatedly, the simplified blood-pressure threshold may have misclassified some younger children: in children below 13 years the 120/80 mmHg cut-off lies closer to the 90th than the 95th age-, sex- and height-specific percentile, so the elevated-blood-pressure component (7.6%) may be slightly over-estimated relative to percentile-based criteria; any such misclassification is expected to be largely non-differential with respect to adiposity, biasing associations towards the null rather than inflating them.

Referral bias and generalisability. Because participants were recruited from the tertiary referral paediatric obesity service of the province's only maternal and child-health institution, the case-mix is likely enriched for more severe obesity, a greater complication burden and more concerned families than would be seen in primary care or community settings. Consequently, the observed ORMC prevalence of 31.3% almost certainly exceeds that of primary-care or community-based populations of children with overweight or obesity. Predictive performance is likewise prevalence- and case-mix-dependent: discrimination, calibration-in-the-large and net benefit would all be expected to shift—typically towards lower event rates and different risk distributions—in lower-risk settings. The model must therefore be re-estimated, or at minimum recalibrated, in primary-care or community cohorts before any wider deployment, and our prevalence and performance estimates should be read as specific to a tertiary referral population.

### 4.5 Clinical and public-health implications

For paediatric obesity clinics in China, our results support three practical steps: (i) systematic metabolic screening—fasting lipids, uric acid, glucose and blood pressure—at the first obesity consultation, as recommended by national consensus (19); (ii) routine incorporation of neck circumference and bioimpedance body fat alongside BMI to improve risk stratification at negligible cost; and (iii) explicit screen-time counselling within family-based weight management. Only if its performance is confirmed in external, ideally prospective multicentre, validation might the parsimonious three-variable LASSO score eventually serve as an intake triage aid to prioritise children for intensified metabolic work-up; the decision-curve findings reported here are exploratory and hypothesis-generating rather than evidence of deployable clinical utility, and in its current form the model is not recommended for clinical decision-making.

## 5 Conclusions

Nearly one-third of children attending an obesity clinic in Northwest China already carried at least one obesity-related metabolic complication, most commonly dyslipidaemia and hyperuricaemia. Adiposity burden captured by BMI, neck circumference and bioimpedance body fat percentage was the dominant, jointly acting predictor domain, and an interpretable LASSO logistic model matched a random forest with modest, internally validated discrimination that requires confirmation in external cohorts. Routine metabolic screening augmented by simple body-composition measures, coupled with screen-time reduction, should be prioritised in paediatric obesity care; the proposed score is hypothesis-generating, and multicentre prospective external validation is a prerequisite to any clinical application.

## Data Availability

The datasets analyzed in this study are not publicly available because they consist of de-identified clinical and questionnaire data protected by institutional privacy and ethical regulations. The de-identified analysis dataset and the complete analysis code are available from the corresponding authors on reasonable request, subject to institutional approval and a signed data access agreement.
